# Dynamic hand and finger load distribution patterns in the first year following surgically treated distal radius fracture

**DOI:** 10.1186/s12891-025-08674-0

**Published:** 2025-05-16

**Authors:** Johannes Karnatz, Christoph Harms, Dagmar-Christiane Fischer, Thomas Mittlmeier

**Affiliations:** 1 Department of Traumatology, Hand and Reconstructive Surgery, Rostock University Medical Center, Rostock, Germany; 2 Department of Pediatrics, Rostock University Medical Center, Rostock, Germany; 3 Department of Pediatrics, University Medical Center Schleswig-Holstein, Kiel, Germany

**Keywords:** Hand function, Load distribution, Grip strength, Range of motion, QuickDASH, Dynamometer, Manugraphy, Distal radius fracture, Surgical treatment

## Abstract

**Background:**

Manugraphy is a relatively young technique for assessing dynamic load distribution when gripping a cylinder. Thereby, dynamic hand grip function is objectivated, providing a more detailed insight than with other clinical assessment tools. Analysis of changes in grip patterns following a distal radius fracture provides a new perspective on documenting the recovery process of this common injury. Our aim was also to investigate the relationships between load distribution during a maximum force grip and other hand function parameters to better understand the implications for follow-up and rehabilitation.

**Methods:**

Assessment of the QuickDASH score, finger and wrist range of motion, grip strength as well as grip load distribution using manugraphy was performed 3, 6 and 12 months following isolated surgically treated distal radius fractures. Using special software, each finger ray as well as the thenar and hypothenar were defined on the digital pressure map and the contribution to the total force was calculated for each region.

**Results:**

After 3 months, 49 patients participated in the follow-up examinations, after 6 months 38 patients and after 12 months 35 patients. When the QuickDASH score decreased significantly, the wrist range of motion and grip strength recovered to more than 90% of the values of the uninjured side within the first year after fracture treatment. The cumulative analysis of the load distribution showed that after 3 months, the thumb and index finger exerted a greater proportion of the total grip strength than did the uninjured hand, whereas the contributions of the thenar and hypothenar were smaller. These changes diminished at 6 and 12 months, respectively. The changes in grip pattern showed significant correlations with grip strength and partly with range of motion of finger and wrist as well as the QuickDASH score.

**Conclusions:**

The dependence between changes in load distribution and different hand function parameters implies the particular additional value and validity of this helpful technique for individual assessment and rehabilitation of hand function. Early detection of persisting imbalances of dynamic load distribution might support clinical decision-making in the postoperative course during rehabilitation.

**Supplementary Information:**

The online version contains supplementary material available at 10.1186/s12891-025-08674-0.

## Background

Fractures of the distal radius represent one of the most common fracture types and might account for considerable permanent functional impairment, such as pain, reduced grip strength and reduced wrist and finger range of motion [1–4]. Furthermore, changes in grip pattern have been reported [5].

Still, cast immobilization is one of the major standard procedures following a successful closed reduction in otherwise stable fracture variants [6, 7]. Surgical treatment is indicated in the case of fracture instability, incongruence of the articular surface as well as severe axial deviation or shortening and it should be performed if reduction is not possible or if nerve and soft tissue damage is imminent [6, 8–10]. Currently, in adults open reduction and volar locking plate fixation is the predominant technique [11].

Outcome measurements have contributed to ameliorating treatment [12]. The evaluation of hand function following a distal radius fracture includes the measurement of clinical parameters as well as the assessment of patient-reported outcome measures (PROMs) [2, 4]. Various types of questionnaires are used to assess and compare subjective ailment in daily tasks and limitations in social or recreational activities, including the Disability of the Arm, and Hand (DASH), its short version QuickDASH and the Patient-Rated Wrist Evaluation (PRWE) [4, 13]. The DASH score correlates well with the QuickDASH and PRWE score [4, 14–18]. Along with the function subscale of the PRWE the QuickDASH questionnaire is recommended to assess hand function following distal radius fractures as the information is comparable to the full DASH questionnaire and the former is completed more frequently [13].

Grip strength measurements are a common procedure in the follow-up of distal radius fractures [4, 13, 19]. The Jamar hand dynamometer is considered the standard instrument, yet it represents a static and global measurement and does not allow for the evaluation of grip patterns [20–22].

Special devices allowed the evaluation of the load distribution between the fingers at certain points of their transverse axis [23, 24]. Over the last two decades, the assessment of grip patterns has evolved and the implementation of electronic processing of multiple sensors has enabled a more detailed evaluation of dynamic load distribution [21, 24–26]. A validation study comparing the manugraphy system (novel, Munich, Germany), which uses cylinders coated with sensor mats, with the Jamar dynamometer showed a consistent correlation in measuring absolute grip strength [21]. The absolute values were greater when measured with the manugraphy system, which could be related to the larger contact and measuring area when using multi-sensor cylinders [21, 27].

In healthy subjects, a similar pressure distribution pattern was found for both hands with acceptable accordance between several studied collectives and could be confirmed by measurements with the manugraphy system [23, 24, 28]. Until now, few measurements have assessed the grip pattern after a fracture or surgery of the hand or forearm [5, 29].

Since manugraphy allows to combine the pressure exerted on multiple sensors to measure the total load or the load on specific areas, our aim was to evaluate changes in grip pattern within the first year of recovery following surgical reconstruction of a distal radius fracture. In addition, we aimed to assess any relationships between load distribution with objective and subjective hand function and handedness to understand its significance in follow-up and functional recovery. The degree of intraindividual loading asymmetry between the injured and the uninjured hand and the imbalance of load transfer during the posttraumatic or postsurgical course might both represent a measure for objectifying the degree of functional deficits and the key for selecting early intervention strategies.

## Materials and methods

### Subjects

We conducted a prospective study of subjective and objective hand function parameters in patients after surgically treated distal radius fracture. Ethical approval has been granted by the medical ethics committee of the University of Rostock (A2024-0123). The participation was voluntary and informed consent was obtained from all subjects involved in the study.

With the help of central medical documentation, all patients who had been surgically treated for a distal radius fracture at the Level I Trauma Center and university hospital between November 2012 and September 2013 were identified. The documented impairments and underlying health conditions were evaluated, and if the patients met the inclusion criteria (Table 1), they were invited for a follow-up examination.

**Table 1:** Inclusion and exclusion criteria

**Inclusion criteria**
• Surgically treated unilateral distal radius fracture • Indication for open reduction and volar plate fixation
• Minimum age of 18 years
• Independent participation possible
**Exclusion criteria**
• Concomitant bone injuries on the same or contralateral arm
• Revision surgery due to dislocation or malunion
• Previous radius fracture within the last 4 years • Neurological impairment with limitations in hand function
• Cognitive deficits with reduced independence
• Severe comorbidity (e.g. cancer or systematic diseases)

### Methods and measurements

The patients were treated with early functional postoperative therapy after removal of a dorsal splint which was applied for the first 3 postoperative days. Appointments at the outpatient department were scheduled three (t_3_), six (t_6_) and twelve (t_12_) months after surgery within the following month. Every study visit included a medical history focusing on impairments and hand function. The 11 items containing QuickDASH questionnaire as a PROM was completed during each visit, covering a score ranging from 0 (no impairment) to 100 (worst impairment) (www.dash.iwh.on.ca) [30, 31].

The maximum extension, flexion, radial deviation and ulnar deviation of the hand in the wrist were measured using a hand-held goniometer. A special ruler was used to measure the distances between the fingertips and the palm when the patient formed a fist (the adapted criterion for pathological hand stiffness was a distance of at least 1 cm for one fingertip) and the maximum span between the tip of the thumb and the little finger [2].

Finally, for both hands, grip strength measurements with spatial resolution of the applied forces were performed with the manugraphy system (novel, Munich, Germany). It uses capacitive sensor mats with a spatial resolution of 2 sensors per square centimeter mounted on cylinders with circumferences of 150 and 200 mm, respectively. The method is highly reproducible with a maximum error < 5% [21]. We used the cylinder with a circumference of 150 mm. Starting with the right hand, the sensor mat-coated cylinder was tightly grasped and, following the standardized “grab” and “release” commands from an audio instruction file, the patients were requested to grip three times with full force for 5 s and to hold the cylinder loose for the interval of 10 s in between.

The chosen diameter of the cylinder and the measurement sequences corresponded to previous studies with the manugraphy system [21, 24]. With the participants sitting upright and their arm bent at 90° at the elbow, the forearm was held horizontally forward and the elbow close to the body [20, 22, 32]. The cylinder was held vertically and a maximum wrist extension of up to 30° was tolerated [22, 28, 33].

The corresponding specific software (pliance^®^, novel, Munich, Germany) was used for the measurements and subsequent offline analysis. From each of the three 5-second intervals of maximum grip strength, the middle 3 s were selected, and the mean of all three was calculated using the cycles^®^ program (novel, Munich, Germany) [34].

Grip patterns were analyzed by manually marking the areas of each finger as well as thenar and hypothenar on a virtual image of the sensor mat, taking into account the shape and elevations of the fingers and palm according to anatomical characteristics [24].

### Statistical analysis

The Shapiro-Wilk test was used to test for a normal distribution. Nonnormally distributed data are presented as the median and range (minimum– maximum), and normally distributed data are presented as the mean ± standard deviation. The Wilcoxon test was used for paired data sets, and the Friedman test was employed to assess longitudinal changes across all three measurements. Group comparisons were performed using the Mann-Whitney *U* test, and correlation coefficients were calculated using the Spearman rank test. The significance tests were two-tailed, and the significance level was set at *p* < 0.05.

For multiple comparative analyses, the measured values of the injured hand were normalized to the values of the healthy hand.

Statistical calculations and graph creation were performed using Prism^®^ software (GraphPad Software, Boston, USA).

## Results

### Subjects

**Fig. 1:**
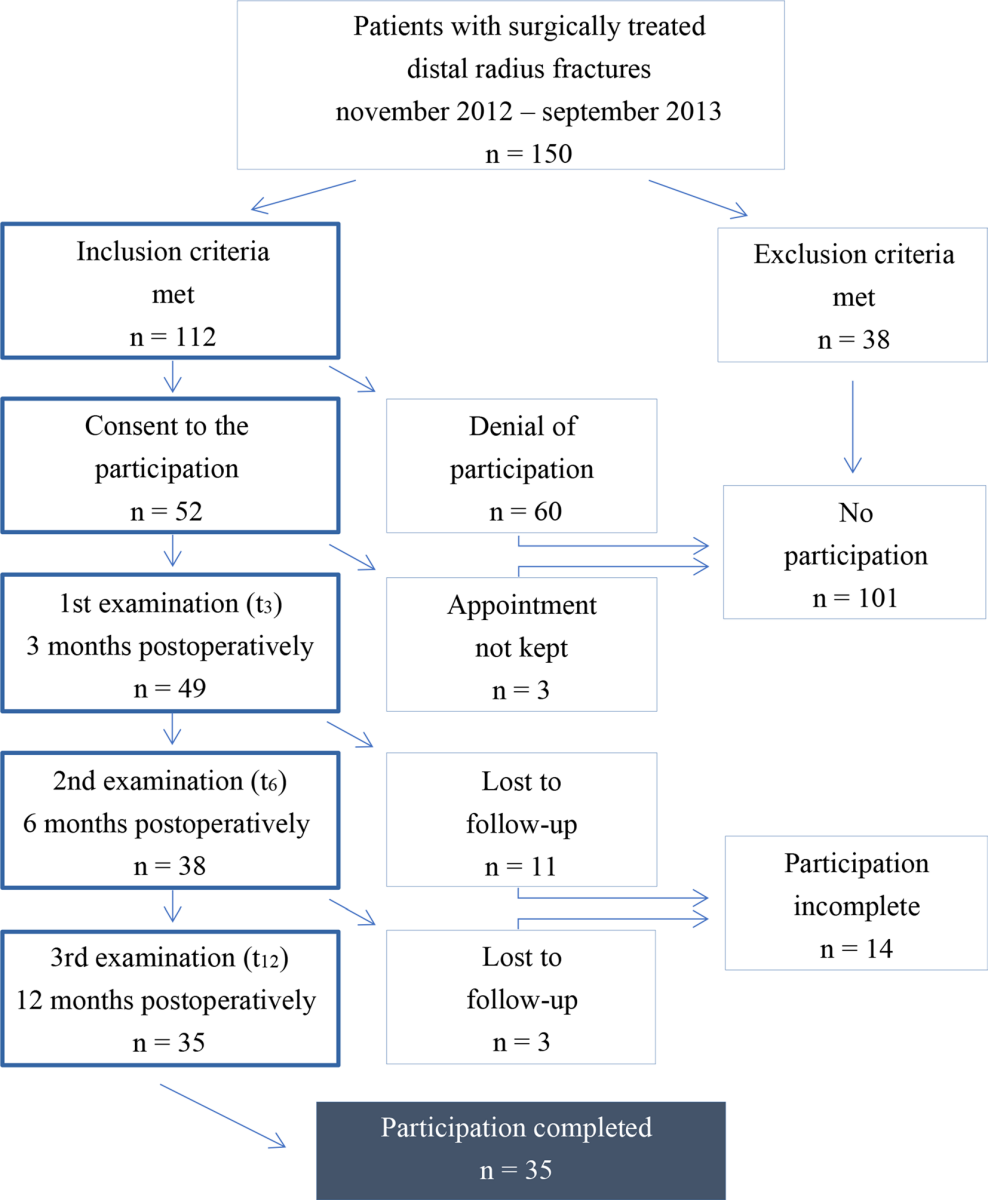
Selection of the participating patients according to inclusion and exclusion criteria and their consent

The numbers of eligible patients and those who provided informed consent and participated in the study are shown in Figure 1. Their characteristics are presented in Table 2.

**Table 2:** Patient characteristics by participation frequency

Participation	Once	Three times
Women / men	41 / 8	30 / 5
Age (years) total / women / men	603 / 635 / 428	603 / 618 / 327
Height (cm) total / women / men	168 / 164 / 180	164 / 164 / 181
Weight (kg) total / women / men	69 / 68 / 92	68 / 66 / 89
BMI (kg/m²) total / women / men	250 / 248 / 268	250 / 248 / 252
Dominant hand (both / left / right)	1 / 2 / 46	1 / 2 / 32
Fracture side (right / left)	24 / 25	18 / 17
AO/OTA classification (A2.2/3 / A3)	13 / 16	12 / 11
AO/OTA classification (B / C)	3 / 17	2 / 10
Plate fixation ^1^/ Radius nail	45 / 4	32 / 3
Temporary external fixator	1	1
Type of accidents/falls: - standing height / black ice / stairs / ≥ 2 m - height / bicycle / ice skating / other sports	28 / 8 / 2 / 2 4 / 3 / 2	22 / 5 / 2 / 1 2 / 1 / 2
Plate / Nail removal within 12 months / 24 months	6 / 10	5 / 9
Malunion / Dislocation	1 / 1	0 / 1
Carpal tunnel syndrome	1	1
Dupuytren’s contracture	2	1
Sensory impairment at 12 months	-	3
Osteoporosis	15	10
Osteoarthritis of the hand	5	4
Rheumatoid arthritis	1	1
Previous distal radius fracture - same side / opposite side	7 4 / 3	5 3 / 2
Diabetes mellitus / Hypertension	3 / 18	3 / 12

^1^volar locking plate (*n* = 43 / 30), dorsal plate fixation in type C fractures (*n* = 2 / 2)

### Determination of finger and hand agility

#### Wrist range of motion

The range of motion of the wrist was still compromised at 3 months (t_3_) in all directions and continued to improve during follow-up. At 12 months (t_12_), flexion and extension were slightly reduced, while radial and ulnar deviation reached the values of the uninjured hand (Table 3).

**Table 3:** Median range of motion relative to the uninjured hand (given in percent)

	t_3_	t_6_	t_12_
Flexion	74.2 ^1,2^ (20.0–100)	84.6 (47.1–107)	92.0 (50.0–108)
Extension	83.3 ^1,2^ (27.3–100)	90,9 (50.0–100)	92,6 (54.5–157)
Radial deviation	80.0 ^2^ (16.7–150)	100 ^1^ (50.0–133)	100 (50.0–167)
Ulnar deviation	87.5 ^2^ (30.0–138)	88.2 ^1^ (33.3–143)	100.0 (62.5–200)

^1^ Denotes significant differences to the following measurement (Wilcoxon test) and ^2^ denotes significant differences between first and last measurements (Wilcoxon test); in each case *p* < 0.05 applies

#### Maximum hand span between the tip of the thumb and the little finger

The median values for the hand span of the injured side minus of the hand span of the uninjured side were − 0.5 cm at 3, 6 and 12 months, respectively, and are presented with their minimum and maximum values in supplementary file 1.

The hand span on the injured side was greater than that on the uninjured side in 3 patients at 3 months, in 5 patients at 6 months and in 4 patients at 12 months.

#### Hand stiffness (minimum distance between the fingertip and palm)

On the injured hand a distance of at least 1 cm between one fingertip and the palm was measured in 18 participants during the complete follow-up, at 3 months in 17 of 49 patients, at 6 months in 7 of 38 patients and at 12 months in 6 of 35 patients. The maximum sum of all distances for a patient was 13 cm at 3 and 6 months and 6 cm at 12 months. One participant had a distance of at least 1 cm for the first time after 6 months.

Five participants with a distance of at least 1 cm between a fingertip and the palm on the injured side also showed a fingertip-to-palm distance of at least 1 cm on the uninjured side. Among them were only two with a distance of more than 1 cm. The maximum sum of distances on the uninjured side was 7 cm at 3 months and 6 cm at 6 and 12 months.

In one patient, a distance of at least 1 cm between one fingertip and the palm was measured on the uninjured side at 3 and 12 months, and in another patient at 6 and 12 months, but in both cases not on the injured side. The first patient mentioned had a previous distal radius fracture on the now uninjured side.

### QuickDASH score

**Fig. 2:**
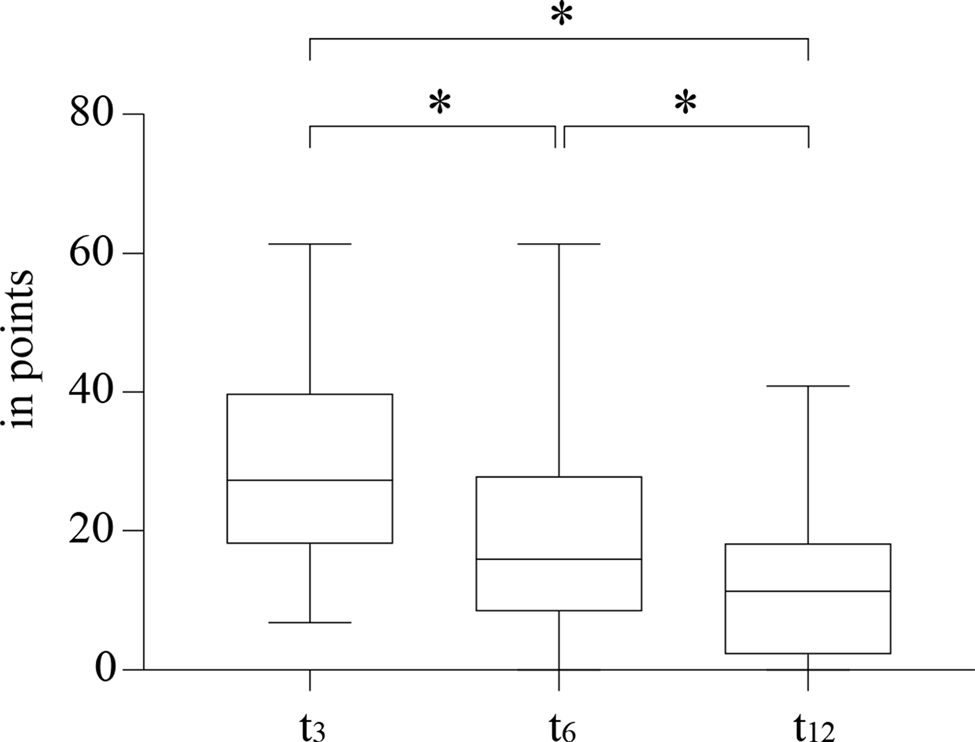
Longitudinal changes in the QuickDASH score, Wilcoxon test: * *p* < 0.0001

The QuickDASH score decreased significantly during follow-up (Fig. 2).

### Grip strength

The absolute grip strength of the injured hand increased significantly during the follow-up.

**Fig. 3:**
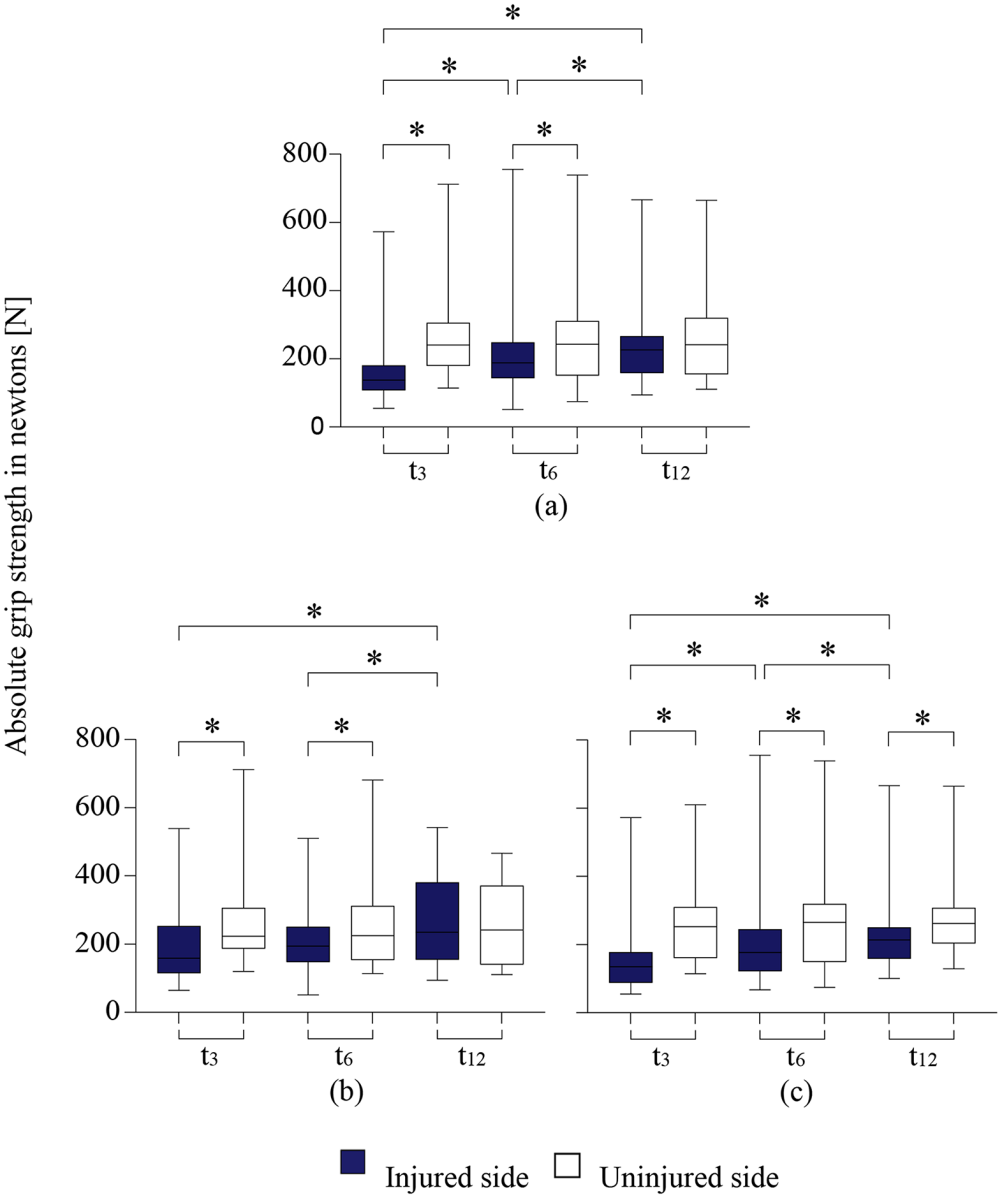
**a-c**: Presentation of the absolute grip strength of all patients (**a**) and their division into those patients with a fracture of the dominant hand (**b**) and a fracture of the nondominant hand (**c**). Superscripts denote significant changes in grip strength and we compared the forces achieved by the injured and uninjured sides during follow-up as well as longitudinal changes for each side. Wilcoxon test: * *p* < 0.05

Figure 3a– c shows the absolute grip strength for all patients and their division into subgroups of those with fractures of the dominant hand and those of the nondominant hand.

Although the absolute grip strength of the injured and uninjured hand differed between the two groups, there were no significant differences.

The grip strength of the injured hand normalized to that of the uninjured hand (relative grip strength) was 62.7% (min. - max.: 17.8–127) at 3 months, 79.5% (min. - max.: 39.7–133) at 6 months and 93.8% at 12 months (min. - max.: 63.9–130). The changes in relative grip strength were significant between all three follow-up measurements (*p* < 0.05).

#### Evaluation of the load distribution when gripping a cylinder

**Fig. 4:**
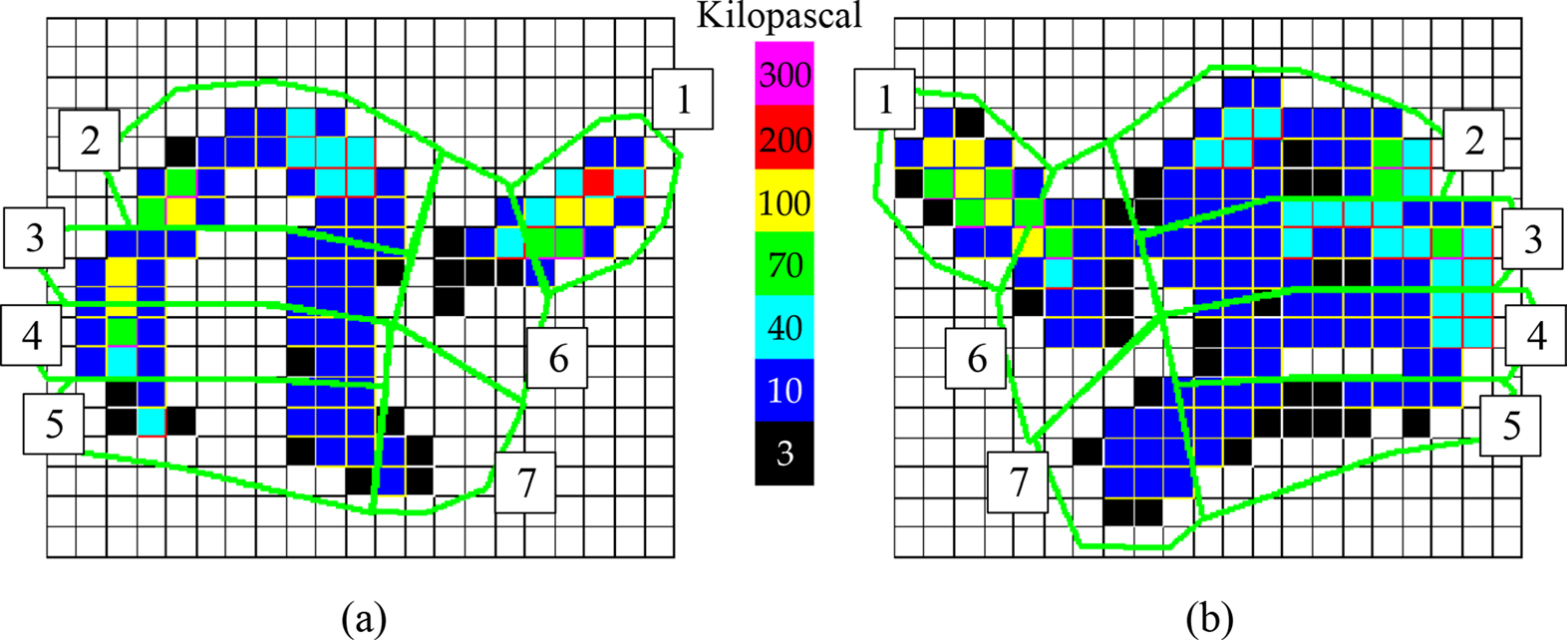
The picture on the left (**a**) shows a typical grip pattern of the injured side 3 months after a surgically treated distal radius fracture and the picture on the right (**b**) shows the uninjured side. For every sensor, the corresponding pressure range is indicated by different colors, as shown by the labelled color column in the middle; (1) 1st finger ray (thumb), (2) 2nd finger ray, (3) 3rd finger ray, (4) 4th finger ray, (5) 5th finger ray, (6) thenar, and (7) hypothenar

A typical example of a change in grip pattern 3 months following a distal radius fracture is presented in Figure 4. These differences diminished after 6 and 12 months.

**Fig. 5:**
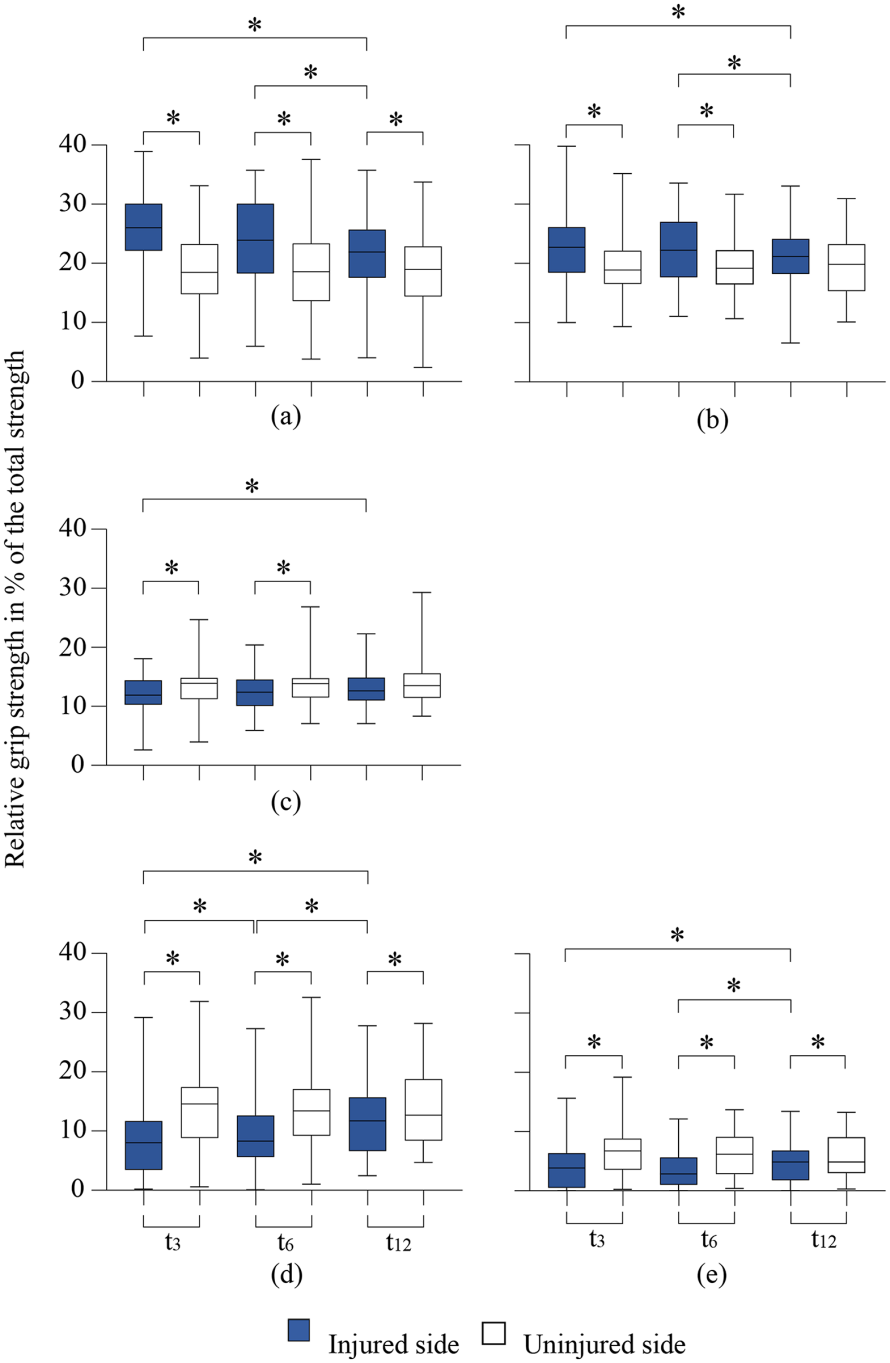
**a - e**: Presentation of the contributions of the individual areas to the total grip strength for the injured and the uninjured side for the areas with significant changes; (**a**) 1st finger ray, (**b**) 2nd finger ray, (**c**) 4th finger ray, (**d**) thenar, and (**e**) hypothenar. Wilcoxon test: * *p* < 0.05

Significant changes in the load distribution of the injured hand compared to the uninjured hand were found for the 1st, 2nd and 4th finger rays, thenar and hypothenar (Fig. 5a– e). No significant changes were found for the 3rd or 5th finger rays.

**Fig. 6:**
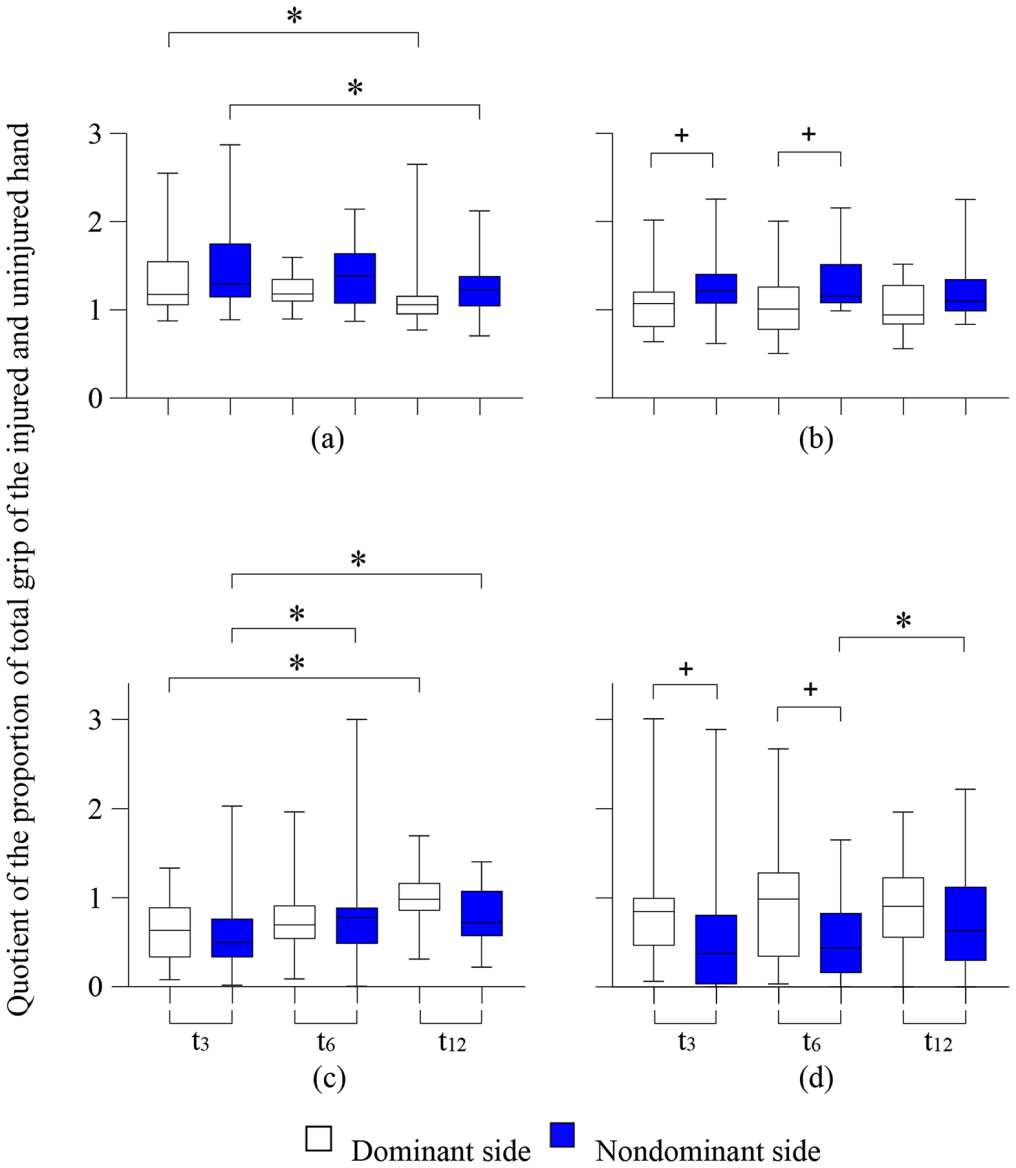
**a - d**: The proportions of total grip strength of the injured side, normalized to the proportion of the uninjured side (which equals a proportion of 1) for the areas with significant changes, separated by handedness; (**a**) 1st finger ray, (**b**) 2nd finger ray, (**c**) thenar, and (**d**) hypothenar. Wilcoxon test: * *p* < 0.05; Mann-Whitney U test: + *p* < 0.05

Figure 6a– d shows the proportion of total grip strength for areas of the injured side, normalized to the corresponding area on the uninjured side, for those with significant changes, differentiated according to handedness. In addition, significant differences between dominant and nondominant hands are indicated.

### Correlation analysis

A correlation analysis was performed between age, QuickDASH score, and grip strength, as well as between each of them and wrist range of motion, reduced hand span, and fingertip-to-palm distances of at least 1 cm (hand stiffness) on the injured side (Table 4).

**Table 4:** Significant correlations between age, QuickDASH score, grip strength and wrist range of motion, hand span as well as hand stiffness

		Age	QuickDASH score	Flexion	Extension	Radial deviation	Ulnar deviation	Hand span	Hand stiffness
t_3_	Age	-							*r* = 0.28
	QuickDASH score		-	*r* = -0.37	*r* = -0.44	*r* = -0.35	*r* = -0.49	*r* = -0.45	*r* = 0.33
	Absolute strength - injured side	*r* = -0.39	*r* = -0.56				*r* = 0.44	*r* = 0.48	*r* = -0.46
	Relative strength		*r* = -0.50	*r* = 0.36				*r* = 0.48	*r* = -0.37
t_6_	Age	-	*r* = 0.41		*r* = -0.34				
	QuickDASH score	*r* = 0.41	-	*r* = -0.45	*r* = -0.70		*r* = -0.49	*r* = -0.39	
	Absolute strength - injured side	*r* = -0.55	*r* = -0.57				*r* = 0.49		*r* = -0.43
	Relative strength		*r* = -0.61	*r* = 0.39	*r* = 0.46		*r* = 0.45	*r* = 0.38	
t_12_	Age	-	*r* = 0.49		*r* = -0.55				
	QuickDASH score	*r* = 0.49	-		*r* = -0.39				
	Absolute strength - injured side	*r* = -0.62	*r* = -0.62		*r* = 0.35		*r* = 0.35		*r* = -0.57
	Relative strength								

For all correlations from Table 4, *p* < 0.05 applies

Grip strength, the QuickDASH score, wrist range of motion, hand span and fingertip-to-palm distances of at least 1 cm (hand stiffness) were significantly correlated with the proportion of total grip strength of the defined hand areas, normalized to the healthy side, as shown in Table 5.

**Table 5:** Significant correlations between load distribution and relative grip strength, QuickDASH score as well as wrist and finger range of motion

		Grip strength	QuickDASH score	Flexion	Extension	Radial deviation	Ulnar deviation	Hand span	Hand stiffness
t_3_	1st finger ray	*r* = -0.34		*r* = -0.51	*r* = -0.37		*r* = -0.30	*r* = -0.40	
	2nd finger ray								
	Thenar	*r* = 0.51				*r* = 0.31		*r* = 0.45	
	Hypothenar	*r* = 0.54	*r* = -0.34	*r* = 0.39					
t_6_	1st finger ray	*r* = -0.32		*r* = -0.45					
	2nd finger ray								
	Thenar	*r* = 0.37		*r* = 0.54	*r* = 0.33			*r* = 0.45	
	Hypothenar	*r* = 0.56							*r* = -0.36
t_12_	1st finger ray	*r* = -0.45				*r* = 0.46			
	2nd finger ray						*r* = 0.58		
	Thenar	*r* = 0.36		*r* = -0.44					
	Hypothenar	*r* = 0.51	*r* = -0.38						*r* = -0.40

For all correlations from Table 5, *p* < 0.05 applies

Additionally, Spearman coefficient calculations revealed a significant correlation between the ulnar deviation and the load of the 4th finger ray, normalized to the uninjured side, at 3 months (*r* = 0.31; *p* < 0.05).

Grip strength on the uninjured side was significantly negatively correlated with age at all follow-up measurements (r -0.49 to -0.62; *p* < 0.001). Negative correlations were found between age and the extension angle of the uninjured side at all follow-up measurements (*r* = -0.33 to -0.43; *p* < 0.05).

## Discussion

Compared with those of the uninjured hand, the grip strength and wrist range of motion of the injured hand improved considerably within the first year following surgical repair of a distal radius fracture and differed by less than 10% after 12 months. The QuickDASH score at 12 months was less than half of the score at 3 months. The evaluation of the load distribution of the injured hand revealed a greater load on the thumb and index finger as well as a lower load on the thenar and hypothenar compared to the uninjured side. Changes in grip pattern significantly correlated with grip strength, QuickDASH score and reduced range of motion of wrist and fingers.

On the uninjured side, grip strength and load distribution did not differ significantly between two follow-up measurements.

A comparison between the values of our study and those of a meta-analysis published by Stinton et al. is presented in Table 6 [4].

**Table 6:** Comparison between the meta-analysis by stinton et al. and our own results

Results of the meta-analysis by Stinton et al. [4]			Own results		
at 3 months	at 6 months	at 12 months	at 3 months	at 6 months	at 12 months
**DASH/QuickDASH score (points)**					
16.3	11.5	7.8	27.3	15.9	11.4
**Flexion (% of uninjured side)**					
76.4	85.4	89.6	74.2	84.6	92.0
**Extension (% of uninjured side)**					
81.6	91.2	94.6	83.3	90.9	92.6
**Radial deviation (% of uninjured side)**					
85.6	92.8	94.4	80.0	100	100
**Ulnar deviation (% of uninjured side)**					
77.2	82.4	91.2	87.5	88.2	100
**Grip strength (% of uninjured side)**					
66.4	77	88.1	62.7	79.5	93.8

Our results confirm the statement of Stinton et al. that the recovery of hand function is largely achieved within the first year following a distal radius fracture [4]. Further improvements were limited [4, 35, 36].

The DASH score from the meta-analysis by Stinton et al. was noticeably different from our QuickDASH values. Some studies have shown a tendency toward slightly higher scores for the QuickDASH questionnaire in direct comparison with the DASH questionnaire [16–18].

Plant et al. analyzed the differences in DASH scores between participants older and younger than 50 years of age following a distal radius fracture [37]. Our results are between those of patients older than 50 years (31.2 points at 3 months, 21.0 points at 6 months and 11.3 points at 12 months) and those younger than 50 years (20.2 points after 3 months, 11.8 points after 6 months and 8.8 points after 12 months) [37].

According to a calculation proposed by Klum et al., a DASH score of 6.5 points for men and 13 points for women could be considered a realistic average at the age of 60 years, similar to our results at 12 months [38].

In addition to the correlations between DASH/QuickDASH score, age, grip strength, flexion, extension, and ulnar deviation, as well as an association with hand stiffness that have been reported, our study showed correlations with radial deviation and maximum hand span [2, 4, 17, 39, 41, 42].

The meta-analysis by Stinton et al. showed a similar range of motion as our results and our finding of a significant correlation between older age and lower extension ability was also evident [4].

An association between reduced wrist mobility and lower grip strength reported by Mifsud and Drew et al. was also evident in our study [43].

In addition to wrist range of motion, Egol et al. reported pathological hand stiffness, defined as a distance of over 1 cm between a fingertip and the palm, in one or more fingers in 19% of patients six months after distal radius fracture [2]. 18% of our patients had a distance of at least 1 cm at six months.

The grip strength reported by the meta-analysis by Stinton et al. was comparable to our own results [4]. Similar findings of 83% at 6 months and 93% at 12 months were obtained by Swedish researchers who compared the grip strength of 90 patients with a mean age of 58 years who had undergone volar plating after distal radius fracture with that of a matched healthy population [44].

Both studies and our own results had in common a correlation between age and grip strength [4, 44]. Furthermore, full-force grip strength corresponds to fracture type (AO/OTA classification), the patient’s height, weight, age and gender as well as somatic and mental health status, but appears to be only slightly related to handedness [4, 38, 45, 50, 51].

Evaluating changes in grip patterns via manugraphy offers a new perspective for objectifying hand function. The precise analysis of every single finger, thenar and hypothenar was not possible with any of the previous dynamometers used [21]. However, the increased load exerted by the thumb and index finger, as in our study, could also be recognized when comparing the maximum pinch and maximum fist grip assessments. Several studies have shown a smaller difference between the injured and uninjured hands in the pinch grip compared to the fist grip in the follow-up of distal radius fractures [52–54].

In addition, a previous and preliminary study on the use of manugraphy performed nine weeks after a distal radius fracture in subjects with a mean age of 67 years showed similar changes to our results, except for the hypothenar [5].

This shift in load distribution toward the thumb and index finger might be related to being mechanically essential for most grasping movements, allowing for quicker recovery from a distal radius fracture [55–58].

Furthermore, it can be assumed that the intrinsic hand muscles, which are mainly involved in the pinch grip, are less affected by a distal radius fracture and the subsequent inflammatory regeneration processes than the extrinsic hand muscles as their tendons are anatomically closer to the fracture site [36, 59, 60].

The reduced mobility of the hand and fingers after a distal radius fracture could be regarded as a limiting factor for flexible adjustment when grasping a cylinder. Being able to involve thenar and hypothenar is related to the grip strength exerted, as the correlations in our study indicate. The reduced loading on the thenar and hypothenar, preferably in the initial post-reconstruction phase, might also be related to pain during a full force grip, as the injured wrist region is directly proximal to them [1, 5].

A possible explanation for the significant differences in grip patterns between injured dominant and nondominant hands at 3 and 6 months might be the preferential use of the dominant hand [61]. This could allow for faster recovery of the dominant hand and thereby explain the smaller changes in grip pattern compared to the nondominant hand shown in our study. Moreover, Wollstein et al. reported better results in several specific hand function tests on the injured side following fractures on the dominant hand compared to fractures on the nondominant hand, yet not for grip strength [62]. The differences in grip pattern between dominant and nondominant hands in healthy subjects appear to be small and were inconsistent in two different studies using manugraphy [24, 63].

The correlations between the changes in grip pattern and grip strength as well as wrist and finger range of motion indicate not only their dependence but also the usefulness of grip pattern analysis to assess limitations in hand function. This approach might not only enhance grip pattern analysis for targeted exercises after an injury and visual feedback during exercise [24]. It also promotes the adoption of this hand function parameter into regular follow-up as it provides an objective assessment of hand function beyond the measurement of grip strength, thus supporting decision-making [24]. Future use might also involve machine learning software analysis to implement the extensive information on hand function into the development of therapeutic strategies [64].

### Limitations and strengths

Our study is limited due to the small number of participants who completed the follow-up and a possible selection bias. The proportion of the total load on the injured and uninjured hand still differed significantly for the thenar and hypothenar one year after fracture reconstruction and since follow-up was limited to the first year following surgically treated distal radius fractures, further development thereafter remains unclear yet.

A strength of our study was the acquisition of multiple clinical hand function parameters and their comparison with manugraphy as well as the shown interconnectivity. Since a substantial number of our findings were reconfirmed by the results given by other authors employing different methods [2, 4, 18, 37, 42–45, 52], the validity of load distribution analysis via manugraphy as a comprehensive technique for assessing hand function could be confirmed.

## Conclusions

The use of manugraphy enables the assessment of grip strength and, at the same time, load distribution as a novel approach to evaluate and objectify the recovery of hand function after injury, promoting a more detailed comprehension of individual changes in load distribution. It enables early analysis and detection of postoperative anomalous grip patterns and supports the development of adequate intervention strategies including training or physiotherapy in order to avoid permanent functional impairments such as reduced range of motion and reduced grip strength.

Further research might be helpful to validate our findings, establish load distribution analysis as a comprehensive hand function parameter, and assess the long-term development of grip patterns along with functional recovery.

## Electronic supplementary material

Below is the link to the electronic supplementary material.

Supplementary Material 1

## Data Availability

The original data presented in the study are partly openly available on the publication server of the University of Rostock at 10.18453/rosdok_id00004260, since the results of all patients who completed all three follow-up assessments were published as a doctoral dissertation. The complete data presented in this study are available on request from the corresponding author.

## Abbreviations

AO/OTA: Arbeitsgemeinschaft für Osteosynthesefragen / Orthopedic Trauma Association
DASH: Disabilities of Arm, Shoulder and Hand (questionnaire)
cm: Centimeter
min: Minimum
max: Maximum
N: Newton
PROMs: Patient-reported outcome measures
PRWE: Patient-Rated Wrist Evaluation (questionnaire)
QuickDASH: Short version of the Disabilities of Arm, Shoulder and Hand (questionnaire)
t_3_: Follow-up assessment after 3 months
t_6_: Follow-up assessment after 6 months
t_12_: Follow-up assessment after 12 months
