# Supplementary material for: Dynamic hand and finger load distribution patterns in the first year following surgically treated distal radius fracture
Source: BMC Musculoskelet Disord. 2025 May 16;26:486. doi: 10.1186/s12891-025-08674-0 (PMC12082916; doi:10.1186/s12891-025-08674-0)
Supplement: Supplementary file 1 — Supplementary Material 1 [file 12891_2025_8674_MOESM1_ESM.docx]

**Electronic supplementary material**

| t_3_ | t_6_ | t_12_ |
| --- | --- | --- |
| Median (min. – max.) | | |
| -0.5  (-3–0.5) | -0.5  (-2–1.5) | -0.5  (-1.5–0.5) |

**Supplementary file 1:** Maximal distance between the tip of the thumb and little finger of the injured side as a difference from the uninjured side (in cm)
